# Regional variations in the diversity and predicted metabolic potential of benthic prokaryotes in coastal northern Zhejiang, East China Sea

**DOI:** 10.1038/srep38709

**Published:** 2016-12-05

**Authors:** Kai Wang, Xiansen Ye, Huajun Zhang, Heping Chen, Demin Zhang, Lian Liu

**Affiliations:** 1 School of Marine Sciences, Ningbo University, Ningbo, 315211, China; 2 Collaborative Innovation Center for Zhejiang Marine High-efficiency and Healthy Aquaculture, Ningbo, 315211, China; 3 Marine Environmental Monitoring Center of Ningbo, SOA, Ningbo, 315012, China; 4 Faculty of Architectural, Civil Engineering and Environment, Ningbo University, Ningbo, 315211, China

## Abstract

Knowledge about the drivers of benthic prokaryotic diversity and metabolic potential in interconnected coastal sediments at regional scales is limited. We collected surface sediments across six zones covering ~200 km in coastal northern Zhejiang, East China Sea and combined 16 S rRNA gene sequencing, community-level metabolic prediction, and sediment physicochemical measurements to investigate variations in prokaryotic diversity and metabolic gene composition with geographic distance and under local environmental conditions. Geographic distance was the most influential factor in prokaryotic β-diversity compared with major environmental drivers, including temperature, sediment texture, acid-volatile sulfide, and water depth, but a large unexplained variation in community composition suggested the potential effects of unmeasured abiotic/biotic factors and stochastic processes. Moreover, prokaryotic assemblages showed a biogeographic provincialism across the zones. The predicted metabolic gene composition similarly shifted as taxonomic composition did. Acid-volatile sulfide was strongly correlated with variation in metabolic gene composition. The enrichments in the relative abundance of sulfate-reducing bacteria and genes relevant with dissimilatory sulfate reduction were observed and predicted, respectively, in the Yushan area. These results provide insights into the relative importance of geographic distance and environmental condition in driving benthic prokaryotic diversity in coastal areas and predict specific biogeochemically-relevant genes for future studies.

Coastal sediment accumulates remnants of anthropogenic and environmental perturbations (i.e., excess chemical pollutants) and serves as a fundamental component in global biogeochemical cycling. Benthic prokaryotes play crucial roles in biogeochemical cycling within marine ecosystems^1^,^2^,^3^. The diversity of benthic prokaryotes has been investigated across various coastal marine ecosystems^4^,^5^,^6^,^7^,^8^,^9^, demonstrating a wide range of environmental drivers shaping benthic prokaryotic communities such as sediment depth^10^, water depth^11^, temperature^12^, ocean currents^5^, sediment texture^8^, salinity^6^, nutrients^13^, organic matter quantity and availability^14^, heavy metals and organic pollutants^7^. Previous studies have suggested that geographic distance also drives variation in benthic prokaryotic communities^15^,^16^. Moreover, random distributions within the entire community and specific functional groups in sediments were commonly reported, as evidenced by a large unexplained variation in the community composition^4^,^17^. However, knowledge about the drivers of prokaryotic diversity in interconnected coastal sediments at a regional scale is still limited. Such information may be beneficial for understanding the relative importance of geographic distance and local environmental condition in shaping the diversity of benthic prokaryotes in coastal ecosystems.

Unraveling the functional potential of prokaryotes is crucial to better understanding their roles in biogeochemical cycling. A growing number of studies have used metagenomic sequencing to reveal the functional potential of prokaryotes in marine sediments, focusing on metabolic pathways involving carbon, nitrogen, and sulfur cycles^13^,^18^,^19^. Alternatively, PICRUSt (Phylogenetic Investigation of Communities by Reconstruction of Unobserved States)^20^, as a bioinformatics tool, has been applied using 16S rRNA gene data to infer the functional profile of microbial communities in human/mammal^21^, soil^20^, seawater and sediment^22^, and stromatolites^23^, providing a glimpse into the metabolic potential of prokaryotic communities, leading to hypotheses on the biogeochemically-relevant genes worth further studies.

The coastal area of northern Zhejiang is an interconnected marine ecosystem in the East China Sea that covers ~200 km, primarily comprising Hangzhou Bay, which is highly influenced by discharges from the Qiantang River and industry^24^; Xiangshan Harbor, a semi-enclosed bay, frequently perturbed by aquaculture^25^; Sanmen Bay, perturbed by booming fishery^26^; the Zhoushan Islands, located at the mouth of Hangzhou Bay and acting as barriers from the estuary to the ocean; coastal eastern Xiangshan, covered by fishery hot spots; and the Yushan Islands, serving as a reserve area^27^. In the present study, we used 16S rRNA gene sequencing and PICRUSt functional prediction to investigate regional variations in the diversity and metabolic functional potential of prokaryotic communities in the surface sediments from above coastal zones to answer three questions: (1) Does geographic distance overwhelm local environmental condition in shaping prokaryotic β-diversity? (2) Do prokaryotic assemblages show biogeographic provincialism across the zones? (3) How does the predicted metabolic potential of prokaryotes shift across the coastal zones?

## Methods

### Study area description, sampling, and physicochemical analyses of sediments

We collected 34 surface sediment samples from 34 stations across six representative coastal zones, including the Yushan Islands (YS), Xiangshan Harbor (XSH), Hangzhou Bay (HZ), the eastern Zhoushan Islands (ZSE), the eastern Xiangshan (XSE), and Sanmen Bay (SM), which are important in ecosystem functioning/service and geographically cover the main marine area of coastal northern Zhejiang (Fig. S1), during a summer cruise (August 15–28, 2013). ArcGIS 10.0 (ESRI, USA) was used to create the sampling map. The sediments were obtained using a box-corer, and the surface subsamples (top 0–5 cm) were collected using a custom-made corer (inner diameter 5 cm); the samples were mixed well in a sterile glass bottle. We selected an empirical criterion (0–5 cm) often used in studies focusing on the surface-layer of sediments^28^,^29^,^30^ to obtain a general pattern of prokaryotic diversity in the surface sediments across the study area. The sediment cores were partly transferred to sterile cryovials and immediately stored in liquid nitrogen prior to DNA extraction. The surface sediment temperature was measured inside the box-corer after the sediments were collected. An additional sample was used to measure gravimetric moisture content (MC). The pH was measured with a SenTix meter (WTW, Germany). The sediment subsamples were partly freeze-dried, ground, homogenized and stored at −20 °C, and the fresh subsamples were stored at 4 °C prior to analyses. Acid-volatile sulfide (AVS) in fresh sediments was volatized using hydrochloric acid, and the amount of sulfide was spectrophotometrically determined based on its reaction with *N,N*-dimethyl-*p*-phenylenediamine to form methylene blue^31^. The sediment texture was measured according to Sperazza *et al*.^32^ using a particle size analyzer (Beckman-Coulter LS 200, USA). The total organic carbon (TOC) and total nitrogen (TN) in the sediments were determined using an Elementar Vario EL II analyzer^33^,^34^. The sediment samples were digested with sulfuric acid to convert total phosphorus (TP) into phosphate, and the phosphate in the extracts was spectrophotometrically determined based on a reaction with vanadium-ammonium molybdate^35^. Heavy metals and arsenic in sediments were measured as previously described^36^, with some modifications in the digestion reagent, comprising nitric acid, perchloric acid, and hydrofluoric acid (5:1:1, *v/v/v*), and subsequently the metals in the extracts were diluted and determined using an inductively coupled plasma mass spectrometry (Agilent 7500 A, USA).

### DNA extraction, 16S rRNA gene amplification, and Illumina MiSeq sequencing

The genomic DNA of sediment samples was extracted using a Power Soil DNA Isolation Kit (MO BIO, USA). The DNA extracts were quantified using a Qubit 2.0 fluorometer (Life Technologies, USA) and subsequently submitted to Novogene Co. Beijing for 16 S rRNA gene amplification, library preparation, and 250 bp paired-end Illumina MiSeq sequencing. Briefly, the 16 S rRNA gene V4 region was amplified using dual-indexed bacterial/archaeal primers 515F-806R^37^ containing adaptor sequences for the MiSeq platform. An aliquot of 10 ng of purified DNA template from each sample was amplified in triplicate in a 30 μL reaction system under the following conditions: denaturation at 98 °C for 1 min, followed by 35 cycles of denaturation at 98 °C for 10 sec, annealing at 50 °C for 30 sec and extension at 72 °C for 30 sec, with a final extension at 72 °C for 5 min. Triplicate polymerase chain reaction (PCR) amplicons of each sample were pooled, purified using a PCR fragment purification kit (Takara, Japan), and subsequently quantified using the Qubit fluorometer. An equimolar amount of PCR amplicons was combined into one pooled sample and sequenced on the Illumina MiSeq platform.

### 16S amplicon processing and analysis

The sequences generated in the present study were deposited in the Sequence Read Archive of DDBJ (http://www.ddbj.nig.ac.jp) and are available under accession number DRA004709. Raw FASTQ files were demultiplexed with QIIME v1.8.0^38^, and the paired reads were joined with FLASH using the default settings^39^. The joined pairs were subsequently quality filtered and analyzed with QIIME. Briefly, the reads were truncated at any site of more than three sequential bases receiving a Phred quality score (Q) < 20, and any read containing ambiguous base calls was discarded, as were reads with <75% (of the total read length) consecutive high-quality base calls. The remaining sequences were chimera assessed using USEARCH^40^. After filtering the chimera reads, the sequences were clustered into operational taxonomic units (OTUs, 97% similarity) using the *pick_open_reference_otus.py* script. The representative sequences were taxonomically assigned against the Greengenes database (13_8_release) and aligned using PyNAST^41^ against a template alignment of the Greengenes core set. A phylogenic tree was generated from the filtered alignment using FastTree^42^. The singletons and Chloroplast sequences were removed, as were the other sequences not assigned to bacteria/archaea. The full dataset (n = 34) contained 1,280,208 clean reads (mean 37,653 reads per sample).

α-Diversity and β-diversity estimates were calculated by even rarefication at 10,000 reads per sample using QIIME, respectively, with multiple indices (number of observed species, Shannon-Wiener index, and phylogenetic diversity) and Bray-Curtis distance between samples. Kruskal-Wallis test was used to compare the variations in prokaryotic α-diversity and sediment physicochemical parameters among zones. We used a Principal Coordinates Analysis (PCoA) plot based on Bray-Curtis distance to visualize the sample clusters and dissimilarity in prokaryotic community composition between zones and subsequently tested the significance of pairwise dissimilarity in community composition between zones with Analysis of Similarity (ANOSIM). Box plots with Multivariate Dispersion Indices (MVDISP) were used to show the dissimilarity and dispersion in community composition between stations in each zone. Simple and partial Mantel tests were used to test the correlations of geographic distance and environmental parameters with prokaryotic β-diversity (variation in community composition), and subsequently a Mantel correlogram was used to examine the significance of distance effect on prokaryotic β-diversity across a subset of different geographic distance scales (classes) among the sampling stations in turn. Pearson correlations between the pairwise matrix of Bray-Curtis distance and geographic distance between stations within each geographic distance class were generated by Mantel tests with 999 permutations. Positive significant correlations (*P* < 0.05) indicate spatial correlation in β-diversity. Distance-based Multivariate Linear Model (DistLM) was performed using the DISTLM_forward3 program^43^ to confirm the environmental drivers shaping community composition and to show their contributions to variation in community composition, where ‘marginal tests’ were used to assess the contribution of each variable alone and ‘sequential tests’ with forward selection were used to evaluate the cumulative contribution of the variables to community variation. Pairwise geographic distances between stations were calculated using the *distance_matrix_from_mapping.py* script with QIIME. MVDISP and ANOSIM were performed in PRIMER v5^44^. Mantel tests and Mantel correlogram were performed in R using ‘vegan’^45^. The dominant discriminant prokaryotic taxa (relative abundance >1% in at least one sample; showing significantly greater relative abundances in one zone compared with those in the other zones) in each coastal zone were identified using Least Discriminant Analysis (LDA) effect size^46^, which employs the factorial Kruskal-Wallis test (*P* < 0.05) to identify taxa with significantly different relative abundances between zones (using one-against-all comparisons). We used a heatmap to visualize the Spearman correlations between highly abundant discriminant taxa (primarily at the family/genus level) from all the zones and sediment environmental variables in R using ‘pheatmap’ package^47^.

### PICRUSt functional prediction

The PICRUSt v1.0.0 pipeline was used to predict the functional potential of prokaryotic communities^20^. Sequences used for PICRUSt prediction were clustered into OTUs (97% similarity) using the *pick_closed_reference_otus.py* script against the Greengenes database (13_5_release) using QIIME. Any reads that did not hit the reference collection were discarded. The rarefied OTU table (8,000 sequences per sample) was used for predicted 16 S rRNA gene copy number normalization using the *normalize_by_copy_number.py* script, and then the metagenome functional profiles were predicted using the *predict_metagenomes.py* script, generating a table of Kyoto Encyclopedia of Genes and Genomes (KEGG) Orthologs (KOs). The resulting table was collapsed at KO level 3 within the pathway hierarchy of KEGG using the *categorize_by_function.py* script. The Nearest Sequenced Taxon Index (NSTI) score, which is the sum of phylogenetic distances for each OTU between its nearest relative with a sequenced reference genome, measured in terms of substitutions per site in the 16S rRNA gene and weighted according to the frequency of that OTU, was used as an indicator for the accuracy of PICRUSt^20^. A PCoA plot based on pairwise Bray-Curtis distances between samples on KEGG level 3 gene ontologies were used to visualize the sample clusters and dissimilarity in the predicted composition of functional gene families between zones. The only ‘Metabolism’ category gene ontologies were filtered from the level 3 output table to generate a second PCoA plot. The metabolism-only gene ontologies were also used for Canonical Correspondence Analysis (CCA), wherein sample ordinations were constrained and co-plotted by environmental parameters with significance using an Analysis of Variance (ANOVA) with 999 permutations (*P* < 0.05). The predicted relative abundances of genes associated with sulfur metabolism (genes with copy number lower than 1,000 in the whole dataset were discarded) were plotted in R using ‘pheatmap’^47^.

## Results

### Variations in key environmental factors

The metadata of the sediment parameters are summarized in Dataset S1. Generally, the AVS concentration and water depth of YS samples were at peak levels across the zones (Fig. S2). However, the sediment temperature of the YS samples was the lowest of all the zones. The sediment texture showed a somewhat spatial pattern, as indicated by the clay and silt contents, particularly in the HZ samples. The TOC and TP showed less overall variation across the zones.

### Prokaryotic α-diversity

No significant difference was observed in prokaryotic α-diversity across the zones as indicated by the number of observed species, Shannon-Wiener index, and phylogenetic diversity (Kruskal-Wallis, all *P* > 0.05; Fig. S3). Moreover, no significant correlation was observed between prokaryotic α-diversity and any of the measured environmental factors (data not shown).

### Prokaryotic community composition

**Figure 1:**
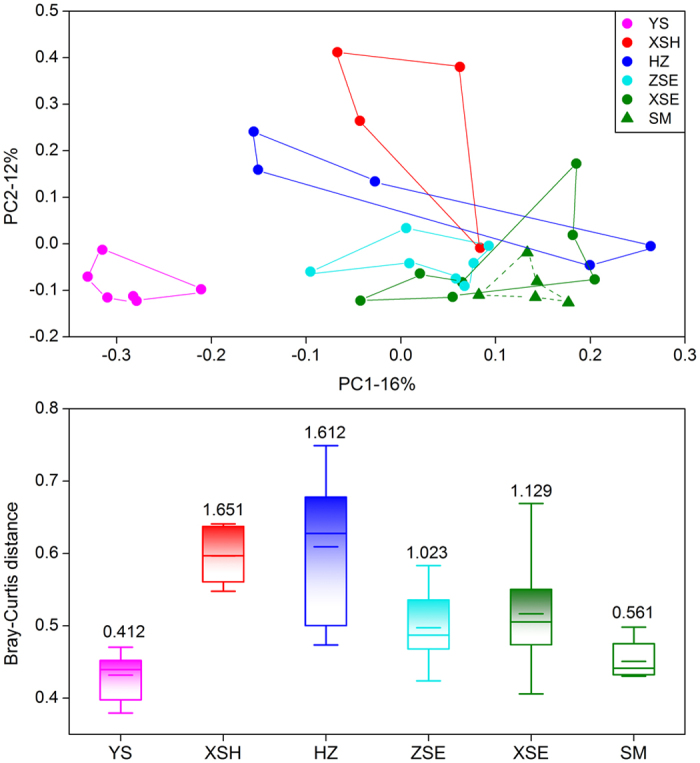
Principal Coordinate Analysis (PCoA) based on Bray-Curtis distance for prokaryotic communities in the surface sediments (upper); Box plots (lower) showing the distance between stations in each coastal zone. The data above the bars are multivariate dispersion indices (MVDISP), and the lines at the top, bottom, and middle of the box correspond to the 75th, 25th, and 50th percentiles (median), respectively. Whiskers at the top and bottom of the box indicate max and minimum values, respectively. The short lines in the boxes indicate the means. YS: Yushan, XSH: Xiangshan Harbor, HZ: Hangzhou Bay, ZSE: eastern Zhoushan Islands, XSE: eastern Xianshan, SM: Sanmen Bay.

The PCoA plot illustrates the dissimilarity of the prokaryotic community composition across the six coastal zones (Fig. 1). We observed two major clusters comprising stations from the YS and the other zones; however, samples from the other five zones also showed somewhat distinct patterns in community composition as indicated by the ANOSIM, except for samples from XSE and SM, which exhibited no significant difference with each other, though prokaryotic compositions in these two zones were significantly different from those in the other zones, respectively (Table 1). Moreover, stations from XSH and HZ showed high within-zone variability according to Bray-Curtis distances and the MVDISP between stations within each zone (Fig. 1).

### Factors driving prokaryotic β-diversity

The results of the Mantel test indicated that geographic distance was correlated with prokaryotic β-diversity (variation in community composition; r = 0.322, *P* = 0.001; Table 2). Temperature, silt, clay, AVS, and water depth were significant individual determinants of community composition, as well as sediment texture and environmental distance (r = 0.208, *P* = 0.027). However, environmental factors showed relatively weaker correlations with prokaryotic β-diversity than geographic distance. Heavy metals, arsenic, TOC, TN, MC, pH, TP, and sand were not significantly correlated with prokaryotic β-diversity. All the environmental drivers (except environmental distance) were correlated with β-diversity when the geographic distance was controlled; however, stronger geographic distance effects on β-diversity were observed with environmental variations controlled compared with environmental effects with geographic distance controlled (Table 2). The Mantel correlogram indicated that spatial correlation in β-diversity was observed at short geographic distance scales among the sampling stations, revealing a patchy distribution of communities with distances up to 36.6 km (Fig. S4). The DistLM showed that two additional factors (TOC and TP) weakly correlated with β-diversity in addition to the other factors identified using Mantel tests (Table 3). Sequential tests with forward selection demonstrated that the environmental factors constrained 30.7% of the community variation.

### Discriminant taxa across coastal zones

**Figure 2:**
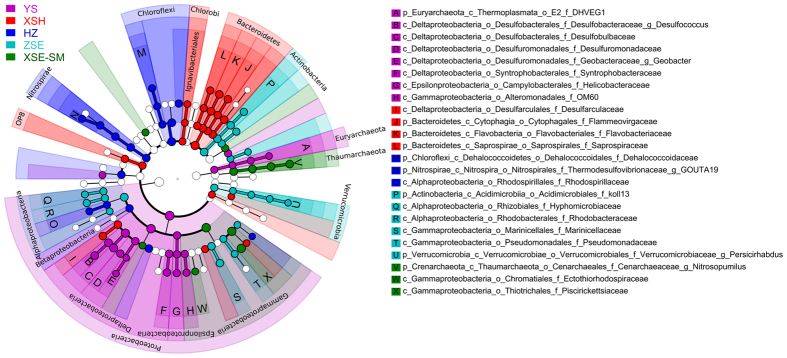
Taxonomic cladogram comparing all samples categorized in the five coastal zones by least discriminant analysis (LDA) effect size. Samples from XSE and SM are tested as the same group according to the non-significant pattern between their compositions as indicated in Fig. 1 and Table 1. Significantly discriminant taxon nodes are colored, and the branch areas are shaded according to the highest ranked group for that taxon. When the taxon was not significantly differentially represented among the sample groups, the corresponding node was colored white. Highly abundant and selected taxa are indicated. For the complete list of discriminant taxa and ranks used to generate this cladogram see Dataset S3. Refer to Fig. 1 for coastal zone abbreviations.

**Figure 3:**
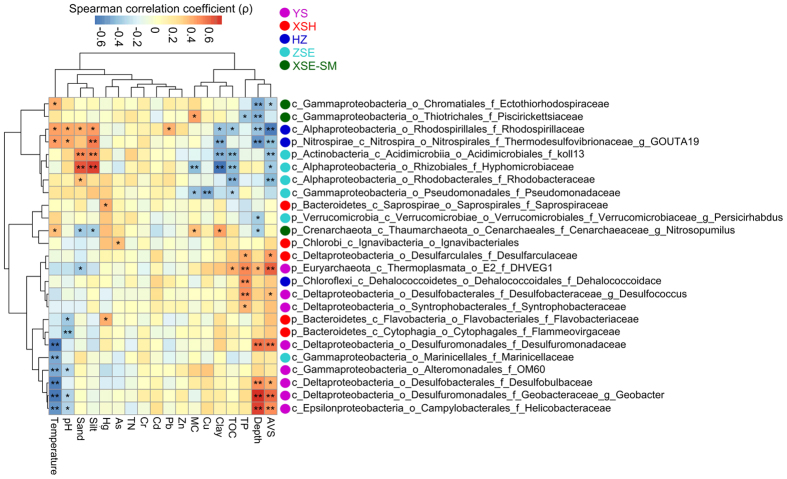
Correlations between highly abundant discriminant taxa (primarily at the family/genus level) of five coastal zones (Fig. 2) and sediment environmental variables. The asterisks in the grids demonstrate significant Spearman correlations as ^**^P < 0.01, ^*^P < 0.05. Refer to Table 2 for variable abbreviations.

Approximately 99% of the clean sequences were classified at the phylum level (Fig. S5). The LDA taxonomic cladogram (Fig. 2) revealed the predominant discriminant taxa of five coastal zones (samples from XSE and SM were tested as the same group according to the non-significant pattern between their compositions as indicated in Fig. 1 and Table 1). δ-Proteobacteria (31.7%, including the families Desulfobacteraceae, Desulfobulbaceae, Desulfuromonadaceae, Geobacteraceae, and Syntrophobacteraceae), ε-Proteobacteria (2.5%, including the family Helicobacteraceae), the candidate γ-Proteobacteria family OM60 (1.6%), and Euryarchaeota (1.0%, including the DHVEG-1 clade) were the most abundant in the YS stations. Bacteroidetes (15.4%, including the families Flammeovirgaceae, Flavobacteriaceae, and Saprospiraceae), the δ-Proteobacteria family Desulfarculaceae (3.9%), and the Chlorobi order Ignavibacteriales (0.72%) were the most abundant in the XSH stations. Chloroflexi (8.4%, including the family Dehalococcoidaceae), Nitrospirae (3.6%, including the family Thermodesulfovibrionaceae), and the α-Proteobacteria family Rhodospirillaceae (1.1%) were the most abundant in the HZ stations. α-Proteobacteria (6.7%, including the families Hyphomicrobiaceae and Rhodobacteraceae), Actinobateria (4.6%, including the koll13 clade), Verrucomicrobia (1.8%, including the genus *Persicirhabdus*), and the two γ-Proteobacteria families Marinicellaceae (3.4%) and Pseudomonadaceae (0.85%) were the most abundant in the ZSE stations. Thaumarchaeota (6.4%, including the genus *Nitrosopumilus*) and the two γ-Proteobacteria families Piscirickettsiaceae (21.0%) and Ectothiorhodospiraceae (0.84%) were the most abundant in the XSE-SM stations. Many discriminant taxa of the YS stations were positively correlated with AVS, some of which were also positively correlated with water depth and/or TP and negatively correlated with temperature (Fig. 3). Overall, the discriminant taxa of the other zones were not well clustered according to their correlation coefficients with measured physicochemical parameters, exhibiting patchy patterns with respect to environmental conditions.

### Predicted metabolic potentials

**Figure 4:**
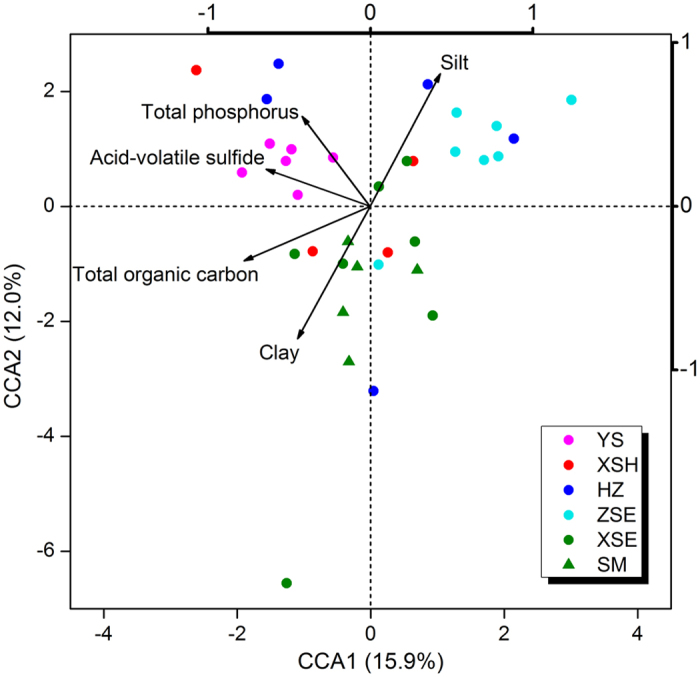
Canonical Correspondence Analysis (CCA) of sediment predicted gene ontologies across sampling stations. PICRUSt predicted function data are based on KOs with only genes classified as “Metabolism” included.

**Figure 5:**
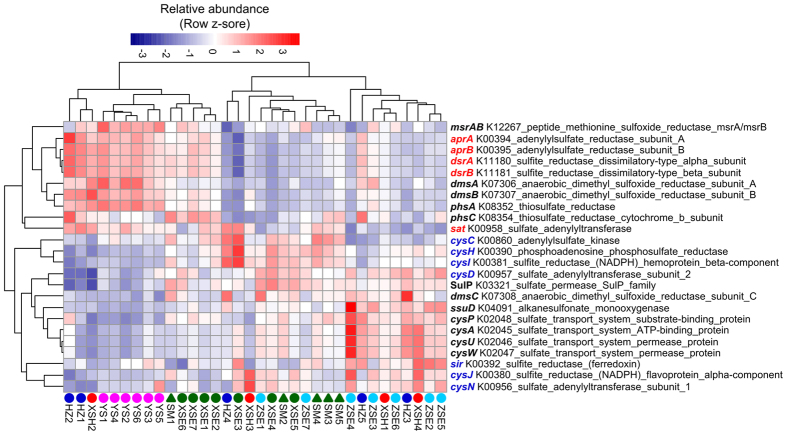
Relative abundances of PICRUSt predicted genes relevant with sulfur (S) metabolism across the coastal zones. The red font indicates the genes relevant with dissimilatory sulfate reduction, and the blue font indicates the genes relevant with assimilatory sulfate reduction.

The NSTI scores of each sample ranged from 0.129 to 0.240, with a mean of 0.186 (Dataset S2). Shifts in functional gene family composition, predicted using PICRUSt, were visualized by three major clusters: YS, ZSE, and XSE-SM; while samples from HZ and XSH showed somewhat random patterns (Fig. S6A). Similar patterns were observed when the Metabolism group of KEGG orthology was examined (Fig. S6B). The Mantel correlation coefficient of the variation in the composition of whole functional gene families with geographic distance (ρ = 0.230, *P* = 0.002) was slightly lower compared with that between the variation in composition of metabolic gene families and geographic distance (ρ = 0.269, *P* = 0.001). However, the variations in taxonomic composition were more strongly correlated with variations in whole functional gene families (Mantel ρ = 0.631, *P* = 0.001) compared with metabolic gene families (Mantel ρ = 0.589, *P* = 0.001). The shifts in metabolic potential were predicted as correlated with AVS, TOC, TP, and sediment texture based on the CCA plot (Fig. 4). Particularly, AVS and TP were positively correlated with the distinct pattern of predicted metabolic potential in the YS stations. Moreover, PICRUSts predicted enrichments in the relative abundance of the genes relevant with dissimilatory sulfate reduction in the YS stations, including genes encoding sulfate adenylyltransferase (*sat*), adenylylsulfate reductase (*aprAB*), and sulfite reductase (*dsrAB*; Fig. 5).

## Discussion

### The relative importance of geographic distance and local environmental condition in prokaryotic β-diversity

Although prokaryotic α-diversity did not show significant variation across the zones, we observed that variations in prokaryotic community composition (β-diversity) between zones were significant, except the variation between XSE and SM. The high within-zone heterogeneity in community composition between samples from XSH and HZ likely reflected the large spatial scale of sampling in HZ and variations in hydrologic conditions in XSH comprising seven distinct hydrologic subzones^48^. The geophysical drivers, such as water depth, temperature, and sediment texture, were also reported in previous studies^8^,^11^,^12^. Besides texture, porosity, as an important sedimentological parameter, was reported to be associated with microbial community structure in the coastal sediments^49^,^50^. Moisture content is known to be used to approximate porosity in the flooded sediments^51^. However, we found that MC was not significantly associated with the variation in prokaryotic composition according to the results of Mantel and DistLM marginal tests, though it contributed 3.9% of variation in DistLM sequential tests. This could be partly explained by the narrow scale of MC in the studied samples (40.3 ± 6.13%, mean ± standard deviation), reflecting that porosity might have less explanatory power compared with other driving parameters. Acid-volatile sulfide is the only chemical driver confirmed using both the Mantel test and DistLM. The vertical gradient of sulfide could be associated with the variation in prokaryotic composition in sediments^52^,^53^. Nevertheless, all the individual environmental drivers were not strongly correlated with prokaryotic β-diversity. Overall, geographic distance was more important in shaping the prokaryotic community composition compared with the measured environmental factors, suggesting a ‘Distance-decay pattern’. However, the Mantel correlogram indicated a patchy distribution of communities across the larger spatial scales, suggesting a somewhat random distribution of prokaryotes with geographic distance. On the other hand, unmeasured abiotic and biotic factors, such as salinity and inorganic nutrients in pore water, predation, and viral infection, might potentially explain the observed variation in prokaryotic composition associated with geographic distance. The large unexplained variation in prokaryotic composition could also reflect potential effects of unmeasured factors and stochastic processes on community assembly. In addition, integrating a relatively large sediment layer (0–5 cm) could cause the vertical heterogeneity in prokaryotic composition and activity associated with biogeochemical gradients, such as the spatial separation of oxygen and sulfide^52^,^54^, to be ignored, thus leading to uncertain variations in prokaryotic composition.

### Discriminant assemblages showed a biogeographic provincialism across the coastal zones

The dominant phyla and proteobacterial classes were similar to those previously reported in coastal sediments^8^,^9^. We observed that Cyanobacteria were not dominant in the sediments, with a low average relative abundance (0.011%, data not shown). This could be partly explained by the limited light-permeability of the turbid waters containing a great amount of suspended particles in the study area overall^27^. Correspondingly, a number of previous works reported that Cyanobacteria were not dominant in the coastal marine sediments^9^,^11^,^55^,^56^. The LDA taxonomic cladogram indicated a biogeographic pattern of prokaryotic assemblages across zones at multiple taxonomic levels. We observed relatively abundant Desulfobacteraceae, Desulfobulbaceae, and Syntrophobacteraceae in the YS stations. Most members of these δ-proteobacterial families are sulfate-reducing bacteria (SRB)^57^. However, some filamentous bacteria belonging to the Desulfobulbaceae likely mediated electrogenic sulfur oxidation at oxic-anoxic interfaces in coastal sediments^54^. The ε-Proteobacteria family Helicobacteraceae was also relatively abundant in this zone. This family is dominated by the *Helicobacter* genus, commonly associated with animal and/or human hosts^58^. However, a number of sulfur-oxidizing species such as *Sulfuricurvum kujiense, Sulfurimonas autotrophica*, and *Sulfurovum lithotrophicum*, classified within this family, have been isolated from sulfur-rich environments including freshwater nature reserves, hydrothermal vents, and deep-sea sediments^58^. Helicobacteraceae were also reported as dominant members in coastal sediments contaminated by organic contaminants^7^. The high AVS concentration and great relative abundances of bacterial groups with potential members relevant with sulfur metabolism suggested that the Yushan area might be suitable for studying the microbial-mediated processes of sulfur metabolism in coastal sediments.

The discriminant assemblages in the XSH stations were dominated by the phylum Bacteroidetes. Members of Bacteroidetes have been implicated in algal organic matter processing^59^ and organic matter degradation in marine sediments^60^,^61^. Since we observed a similar level of TOC in XSH as in the other zones, the availability and composition of organic matter should be estimated in future studies to examine the hypothesis that the organic matter in the XSH sediments could be more favorable for Bacteroidetes. The phyla Chloroflexi and Nitrospirae were relatively abundant in the HZ stations. The discharge of the Qiantang River led to a high concentration of inorganic N in the waters of this zone^27^. Given the shallow water depth, we inferred that the pore water of these sediments might contain a great amount of inorganic N. Some members of Chloroflexi and Nitrospirae catalyze the second step of nitrification^62^. The dominance of these taxa might be consistent with their roles in N metabolism. The Chloroflexi family Dehalococcoidaceae contains members with a dehalogenation complex^63^. A high level of halogenated organic pollutants in the sediments of this zone has been reported, resulting from the industrialization of the surrounding cities^64^. We inferred that the higher relative abundance of Dehalococcoidaceae might be associated with halogenated pollutants. Notably, the above potential relationships should be verified with more details from biogeochemical profiles in future studies. The discriminant assemblages in the ZSE stations were dominated by α-Proteobacteria and Actinobacteria. The major Actinobacteria clade koll13 and the α-Proteobacteria family Hyphomicrobiaceae were positively correlated with the contents of sand and silt in the sediments. These findings might be partly explained by the mycelial structure of members of Actinobacteria and Hyphomicrobiaceae, which colonize more easily in sand/silt-rich (larger particle size) sediments. The discriminant assemblages in the XSE-SM stations were dominated by Thaumarchaeota and the γ-Proteobacteria family Piscirickettsiaceae. As a major member of Thaumarchaeota in this zone, the genus *Nitrosopumilus (N. maritimus*) includes ammonia-oxidizing archaea^65^. One of the Piscirickettsiaceae genera *Piscirickettsia* includes important fish pathogens, such as *P. salmonis*^66^, whose potential presence might be associated with the highly active fishery and aquaculture in these two zones.

Overall, we did observe discriminant assemblages across the coastal zones, suggesting some biogeographic provincialism of benthic prokaryotes in this marine ecosystem. Some of the assembly patterns in each zone might reflect the measured environmental factors and/or reported local features. However, most of the discriminant taxa were poorly associated with the measured factors, and the relationships between local environmental conditions and discriminant taxa did not cluster discriminant taxa from each zone well, suggesting more sufficient biogeochemical data should be collected to obtain a better understanding of the biogeographic provincialism of prokaryotes in future studies.

### Alternation in predicted metabolic potential across coastal zones

The PICRUSt has been used to accurately predict the functional profiles of the human microbiome and soils^20^. Although the accuracy of PICRUSt generally decreased with increasing NSTI score, reliable results were generated from a dataset of soil samples with a mean NSTI score of 0.17; however, environments containing much unexplored diversity, such as the Guerrero Negro hypersaline microbial mats, showed a markedly low accuracy with a mean NSTI score of 0.23^20^. In the present study, the marine sediments also contained much unexplored diversity (lost in the closed reference OTU picking procedure), with a mean NSTI score of 0.186, suggesting that the results are sketchy and should be carefully interpreted. Thus, we explored the variation in the composition of functional gene families at a higher and more general level of KEGG. We observed that the composition of whole functional gene families was distinct in YS, ZSE, and XSE-SM stations, while samples from XSH and HZ showed higher within-zone variability, similar to the pattern in taxonomic composition. However, the overall variation in functional gene composition between stations within or among zones was much lower compared with that in the taxonomic composition comparisons, indicated by the one-order magnitude difference in the scale of PCoA axes, reflecting the Bray-Curtis distance between samples. This converging trend in the predicted functional structure of communities might be partly explained by the functional redundancy hypothesis^67^ and the sketchy results generated using PICRUSt.

Overall, the environmental factors driving the predicted composition of metabolic gene families are similar to those driving the taxonomic composition. Because we observed AVS as a most important chemical factor correlated with metabolic gene family composition, we specifically investigated the relative abundances of the genes relevant with sulfur metabolism across the coastal zones. The greater relative abundances of genes relevant with dissimilatory sulfate reduction were predicted in the YS stations compared with other zones (except two HZ stations and one XSH station). Particularly, *dsrAB* are commonly used as diagnostic markers in ecological studies of sulfite- and sulfate-reducing microorganisms^68^. In addition, SRB depend on a *dsrAB*-type dissimilatory sulfite reductase as a crucial part of the enzymatic system for sulfate respiration^69^. Most *dsrAB* sequences from Svalbard and Greenland sediments were affiliated with Desulfobacteraceae, Desulfobulbaceae, and Syntrophobacteraceae^68^, corresponding to the dominance of δ-proteobacterial SRB in the YS stations and other coastal surface sediments^70^,^71^. Sulfate reduction is the dominant pathway for organic matter mineralization in coastal marine sediments, leading to the accumulation of sulfide in deeper sediments^72^. This process could be affected by sulfate availability, methane emission, and labile organic matter^73^. Labile organic matter has been reported associated with SRB, which serve as electron donors in sulfate reduction^19^,^74^. However, according to the metabolic flexibility of SRB, many of these species could grow as syntrophs in cooperation with methanogens in sulfate-free sediments^73^. Although the enrichment in the relative abundances of SRB and the genes relevant with dissimilatory sulfate reduction was observed and predicted in the Yushan stations, respectively, the lack of sulfate data restricts the evaluation of sulfate reduction potential. Moreover, these SRB cells could be inactive and dormant in communities without active expression of relevant genes. One the other hand, extracellular DNA from dead cells containing amplifiable ribosomal genes in sediments could lead to biases in the estimation of microbial diversity and abundance^75^.

In the present study, the relative abundances of taxa and genes were observed and sketchily predicted using 16S rRNA gene amplicon sequencing and PICRUSt, respectively, without the quantitative estimation of specific taxa and accurate functional genes in an absolute framework. Therefore, these preliminary results should be confirmed and extensively investigated in future studies to better link taxonomy and biogeochemical functions via sufficient geochemical analyses such as concentrations of sulfur species, availability and composition of organic matter, and methane emissions coupled with more quantitative and reliable microbiological analyses such as fluorescent *in situ* hybridization (FISH) for taxon abundance, as well as internal standard added quantitative PCR (qPCR)/metagenomic sequencing and reverse transcription-qPCR/metatranscriptomic sequencing to examine the abundance and expression of functional genes, respectively.

## Conclusion

The findings of the present study provide insights into the regional variations in diversity and predicted metabolic potential of benthic prokaryotic communities in one of the most important coastal areas of China. The results demonstrated the relative importance of geographic distance and environmental drivers in shaping the taxonomic composition of benthic prokaryotes in an interconnected coastal marine ecosystem and generally predicted a regional pattern of metabolic gene family composition, suggesting future hypotheses for more quantitative and reliable studies focusing on the specific taxa and functional genes of interest.

## Additional Information

**How to cite this article**: Wang, K. *et al*. Regional variations in the diversity and predicted metabolic potential of benthic prokaryotes in coastal northern Zhejiang, East China Sea. *Sci. Rep.* **6**, 38709; doi: 10.1038/srep38709 (2016).

**Publisher's note:** Springer Nature remains neutral with regard to jurisdictional claims in published maps and institutional affiliations.

## Supplementary Material

Supplementary Figures

Supplementary Dataset s1

Supplementary Dataset s2

Supplementary Dataset s3

## Figures and Tables

**Table 1:** One-way Analysis of Similarity (ANOSIM) based on Bray-Curtis distance for prokaryotic communities in surface sediments between coastal zones (Global R = 0.587, *P* < 0.001, 999 permutations).

	YS	XSH	HZ	ZSE	XSE
XSH	**0.893**, *P* = 0.005				
HZ	**0.781**, *P* = 0.002	**0.456**, *P* = 0.016			
ZSE	**0.905**, *P* = 0.001	**0.552**, *P* = 0.021	**0.558**, *P* = 0.003		
XSE	**0.799**, *P* = 0.001	**0.466**, *P* = 0.024	**0.441**, *P* = 0.013	**0.223**, *P* = 0.014	
SM	**1.000**, *P* = 0.002	**0.738**, *P* = 0.008	**0.468**, *P* = 0.008	**0.716**, *P* = 0.001	0.085, *P* = 0.234

Bold R values present significant differences (*P* < 0.05); YS: Yushan, XSH: Xiangshan Harbor, HZ: Hangzhou Bay, ZSE: eastern Zhoushan Islands, XSE: eastern Xianshan, SM: Sanmen Bay.

**Table 2:** Simple Mantel test demonstrating the correlations of sediment environmental variations (Euclidean distance) and geographic distance with the variation in prokaryotic community composition (based on Bray-Curtis distance, 999 permutations).

	Simple		Controlled by	Partial	
	r	*P*		r	*P*
Geo_distance	**0.322**	0.001	Temperature	**0.274**	0.003
Geo_distance	—	—	Silt	**0.254**	0.001
Geo_distance	—	—	Texture	**0.256**	0.002
Geo_distance	—	—	Clay	**0.259**	0.003
Geo_distance	—	—	AVS	**0.311**	0.002
Geo_distance	—	—	Env_distance	**0.285**	0.002
Geo_distance	—	—	Water_depth	**0.307**	0.002
Temperature	**0.278**	0.001	Geo_distance	**0.220**	0.002
Silt	**0.271**	0.003	Geo_distance	**0.182**	0.025
Texture	**0.262**	0.004	Geo_distance	**0.172**	0.035
Clay	**0.256**	0.001	Geo_distance	**0.166**	0.039
AVS	**0.224**	0.036	Geo_distance	**0.207**	0.042
Env_distance	**0.208**	0.027	Geo_distance	0.140	0.096
Water_depth	**0.199**	0.025	Geo_distance	**0.173**	0.039
Cd	0.175	0.068	Geo_distance	0.168	0.092
TOC	0.142	0.078	Geo_distance	0.063	0.243
TN	0.089	0.188	Geo_distance	0.084	0.211
MC	0.063	0.170	Geo_distance	−0.085	0.916
Cr	0.047	0.321	Geo_distance	0.044	0.318
pH	0.017	0.392	Geo_distance	0.019	0.388
TP	0.009	0.431	Geo_distance	0.029	0.348
As	0.001	0.486	Geo_distance	−0.010	0.542
Hg	−0.001	0.472	Geo_distance	0.014	0.416
Zn	−0.010	0.514	Geo_distance	0.034	0.343
Sand	−0.023	0.569	Geo_distance	−0.098	0.832
Pb	−0.045	0.689	Geo_distance	−0.061	0.740
Cu	−0.052	0.701	Geo_distance	−0.024	0.590

Partial Mantel test demonstrating the correlation of geographic distance with variation in community composition controlled by significantly correlative environmental variables obtained from simple Mantle tests and those of environmental variables controlled by geographic distance. Data in bold indicate significant correlations (*P* < 0.05). Geo_distance: geographic distance; env_distance: environmental distance (Euclidean distance); AVS: acid-volatile sulfide; TOC: total organic carbon; TN: total nitrogen; MC: moisture content; TP: total phosphorus.

**Table 3:** Distance-based multivariate linear model of the variability of prokaryotic community against sediment environmental variables with 999 permutations.

Marginal tests				
Variable	pseudo-F	*P*	Explained prop.	
Water_depth	3.663	0.001	**0.103**	
Temperature	3.470	0.001	**0.098**	
Clay	2.240	0.006	**0.065**	
Silt	2.233	0.004	**0.065**	
AVS	2.201	0.005	**0.064**	
TOC	1.741	0.018	**0.052**	
Sand	1.636	0.040	**0.049**	
TP	1.594	0.030	**0.047**	
pH	1.487	0.069	0.044	
MC	1.465	0.077	0.044	
Hg	1.194	0.219	0.036	
Cd	1.072	0.334	0.032	
Pb	1.038	0.368	0.031	
TN	0.969	0.464	0.029	
As	0.887	0.589	0.027	
Cr	0.827	0.726	0.025	
Cu	0.827	0.723	0.025	
Zn	0.724	0.887	0.022	
**Conditional (sequential) tests**				
**Variable**	**pseudo-F**	***P***	**Explained prop.**	**Cumulative prop.**
Water_depth	3.663	0.001	**0.103**	**0.103**
Clay	2.434	0.001	**0.065**	**0.168**
TOC	2.124	0.002	**0.055**	**0.223**
Temperature	1.774	0.015	**0.045**	**0.268**
MC	1.574	0.033	**0.039**	**0.307**
AVS	1.367	0.076	0.033	0.340
Cd	1.194	0.178	0.029	0.369
Hg	1.128	0.253	0.027	0.396
pH	1.044	0.369	0.025	0.422
Sand	1.115	0.294	0.027	0.448
TN	1.025	0.393	0.025	0.473
TP	0.936	0.566	0.023	0.495
Zn	1.004	0.463	0.024	0.520
Cu	1.020	0.447	0.025	0.544
Pb	0.976	0.494	0.024	0.567
As	0.866	0.654	0.021	0.588
Cr	0.848	0.661	0.021	0.609

Marginal tests: each variable was analyzed individually (ignoring other variables); Sequential tests: forward selection of variables, where the proportion of variation explained by each variable added to the model was conditional on the variables previously included in the model. Data in bold present significant correlations (*P* < 0.05); Refer to Table 2 for variable abbreviations.
