# Supplementary material for: Regional variations in the diversity and predicted metabolic potential of benthic prokaryotes in coastal northern Zhejiang, East China Sea
Source: Sci Rep. 2016 Dec 5;6:38709. doi: 10.1038/srep38709 (PMC5137025; doi:10.1038/srep38709)
Supplement: Supplementary Figures [file srep38709-s1.pdf]

Supplementary Figures

Regional variations in the diversity and predicted metabolic potential of benthic prokaryotes in coastal northern Zhejiang, East China Sea

Kai Wang, Xiansen Ye, Huajun Zhang, Heping Chen, Demin Zhang & Lian Liu

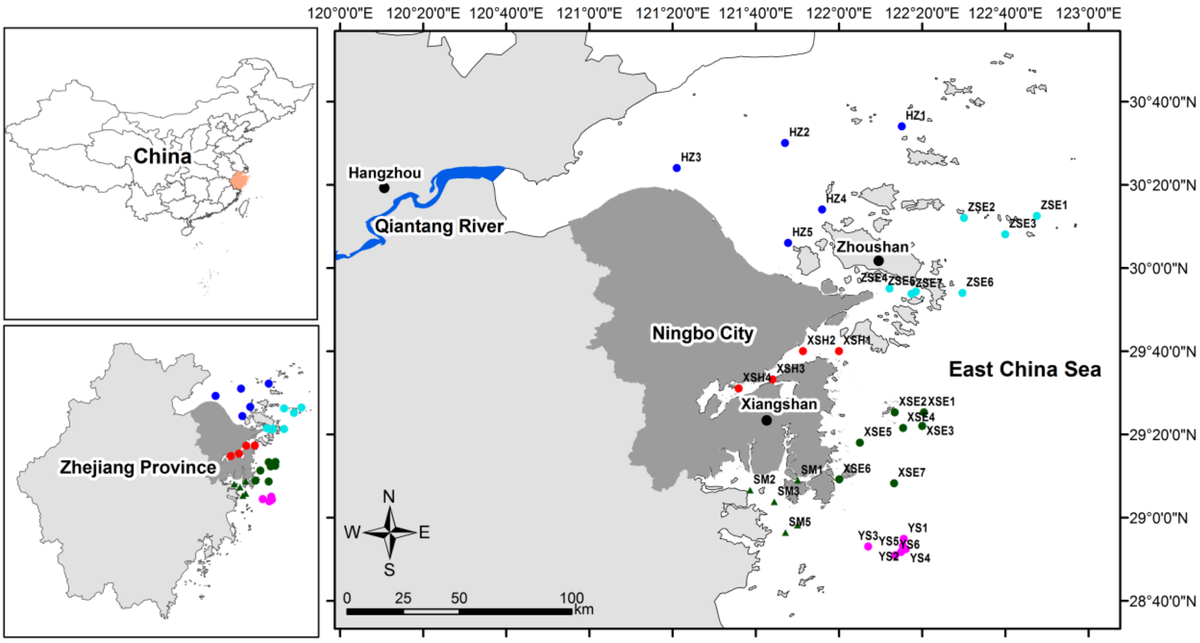

Figure S1. Map of the sampling stations from the six coastal zones in the northern Zhejiang, East China Sea. YS: Yushan, XSH: Xiangshan Harbor, HZ: Hangzhou Bay, ZSE: eastern Zhoushan Islands, XSE: eastern Xiangshan, SM: Sanmen Bay. The map was created using ArcGIS 10.0 (https://www.arcgis.com).

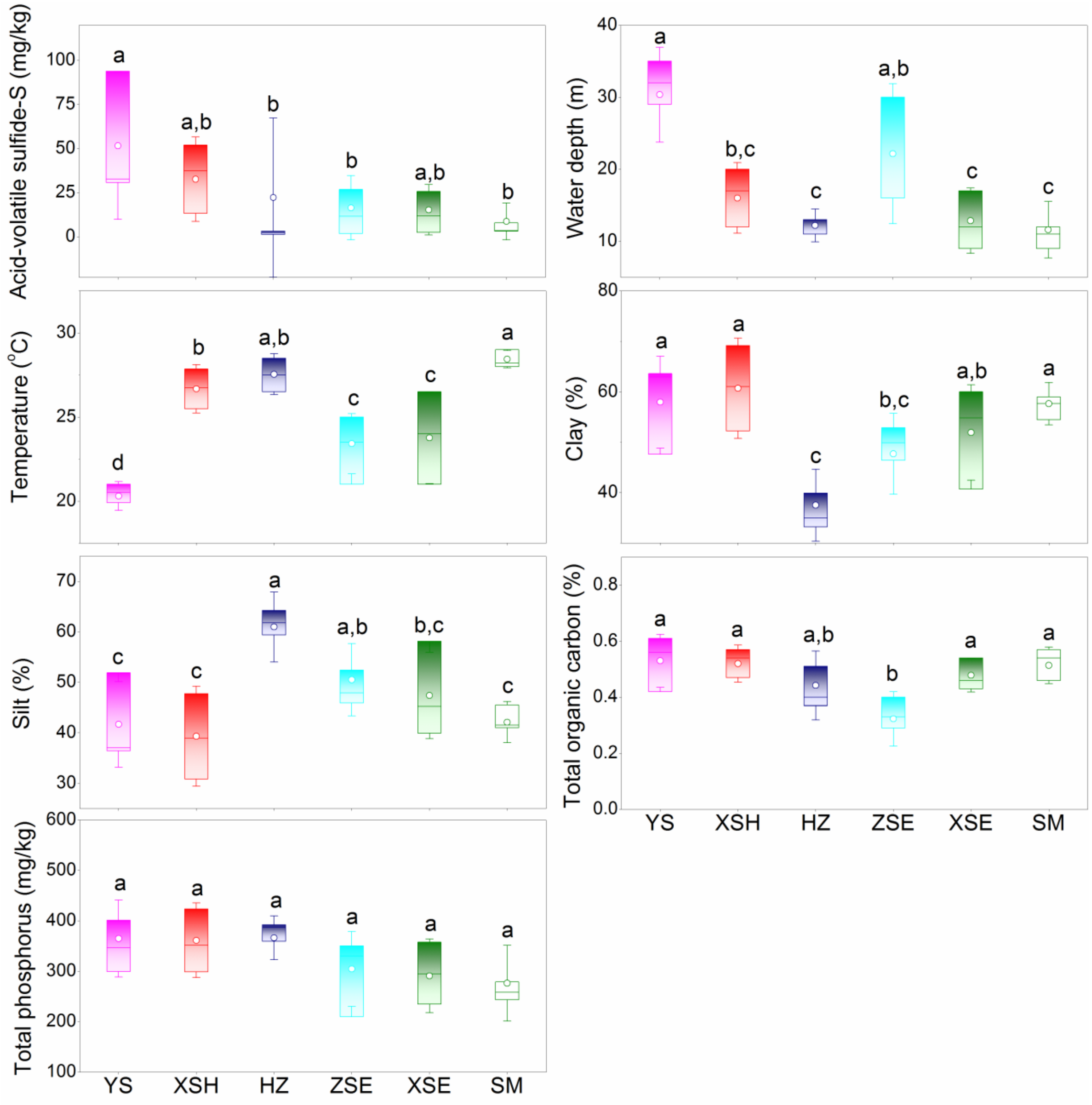

Figure S2. Boxplots illustrating the variations of key environmental factors in sediments across six coastal zones. The lines at the top, bottom, and middle of the box correspond to the 75th, 25th, and 50th percentiles (median), respectively. Whiskers indicate ± standard deviation. The white dots indicate the means. Data sets sharing a letter above the bars are not significantly different from each other, whereas two data sets without a letter in common are significantly different (multiple comparison test after Kruskal-Wallis, $P < 0.05$).

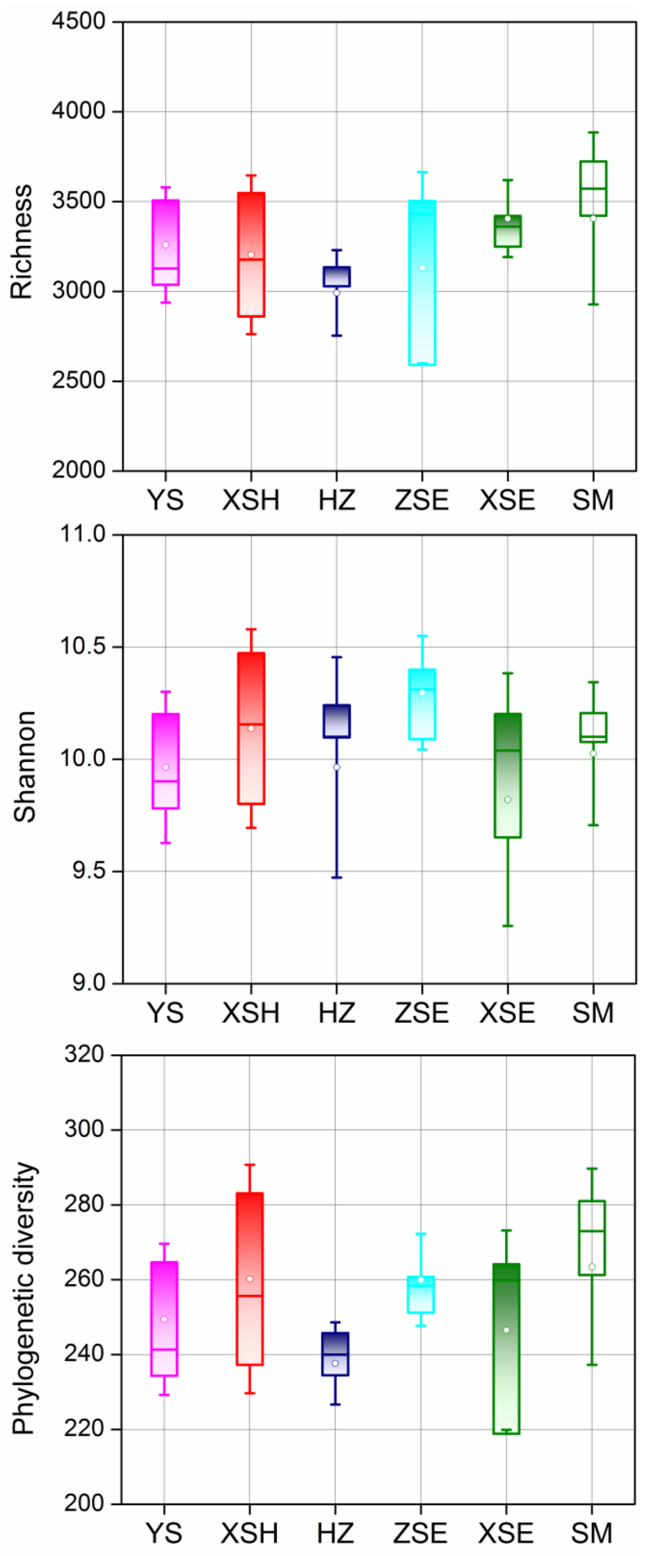

Figure S3. α-Diversity of prokaryotic community across six coastal zones. The lines at the top, bottom, and middle of the box correspond to the 75th, 25th, and 50th percentiles (median), respectively. Whiskers indicate ± standard deviation. The white dots indicate the means.

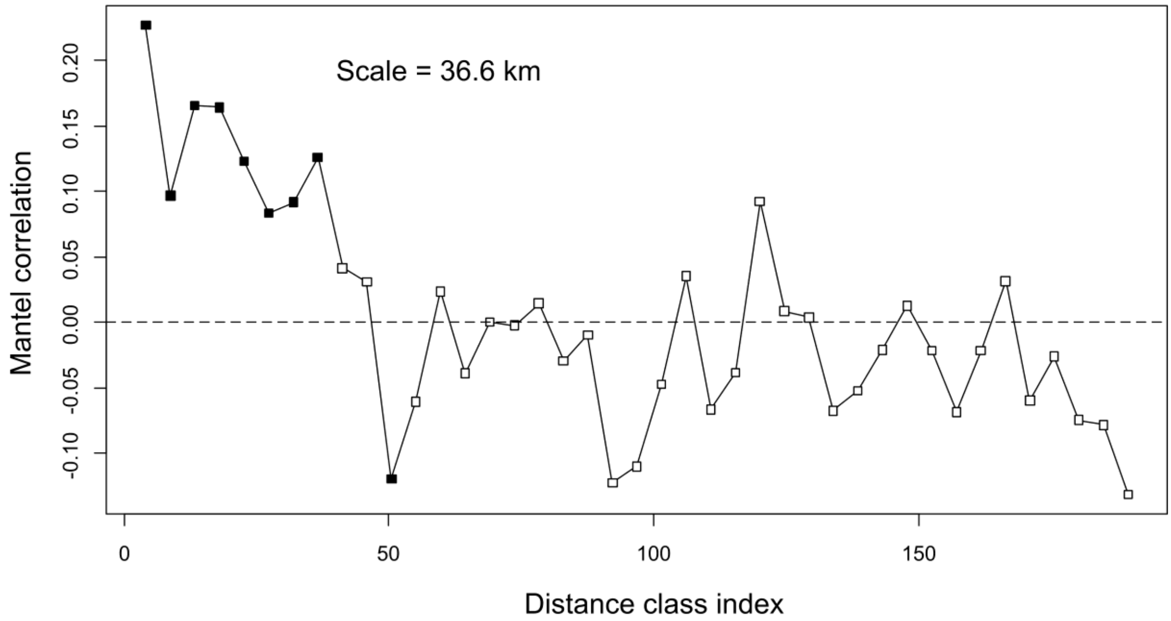

Figure S4. Mantel correlogram demonstrating the significance of distance effect on prokaryotic β-diversity across a subset of different geographic distance scales (classes) among the sampling stations. Pearson correlations between the pairwise matrix of Bray-Curtis distance and geographic distance between stations within each geographic distance class were generated by Mantel tests with 999 permutations. Significant correlations ($P < 0.05$, solid squares with positive values) indicate spatial correlation in β-diversity, observed at short geographic distance scales among the sampling stations.

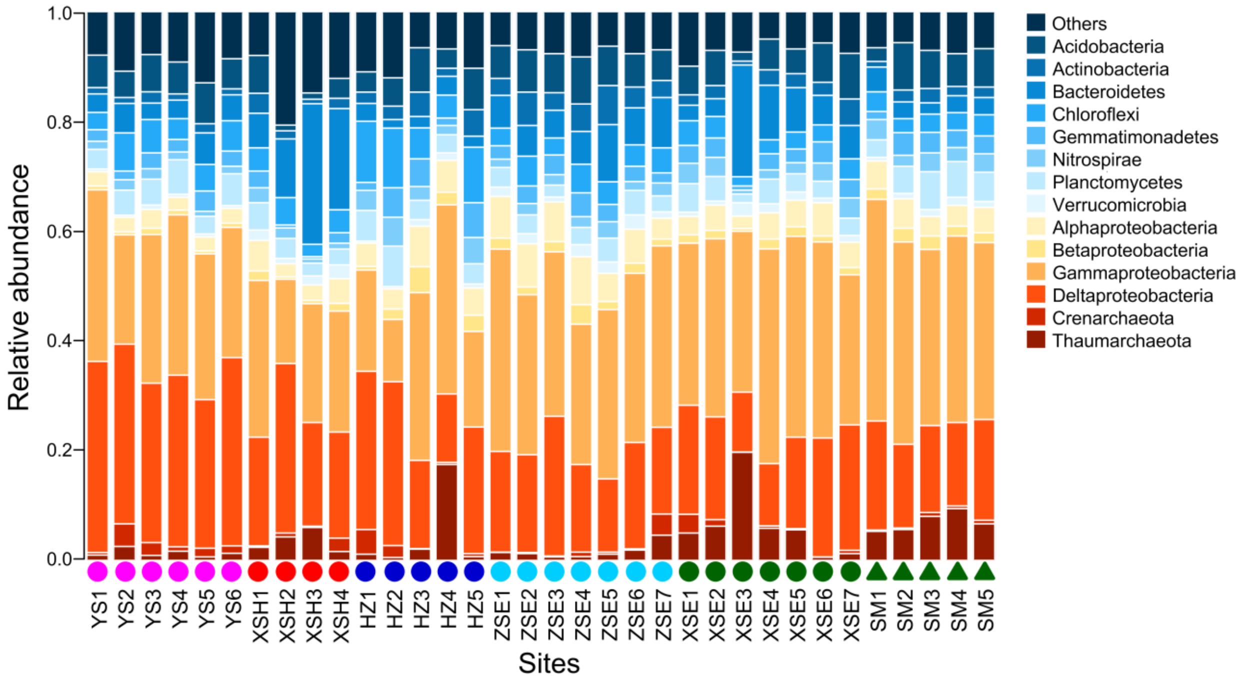

Figure S5. Relative abundances of the dominant phyla (average relative abundance >1%; phylum *Proteobacteria* is grouped at the class level; we filtered *Thaumarchaeota* reads out of the phylum *Crenarchaeota* as classified in the Greengenes database).

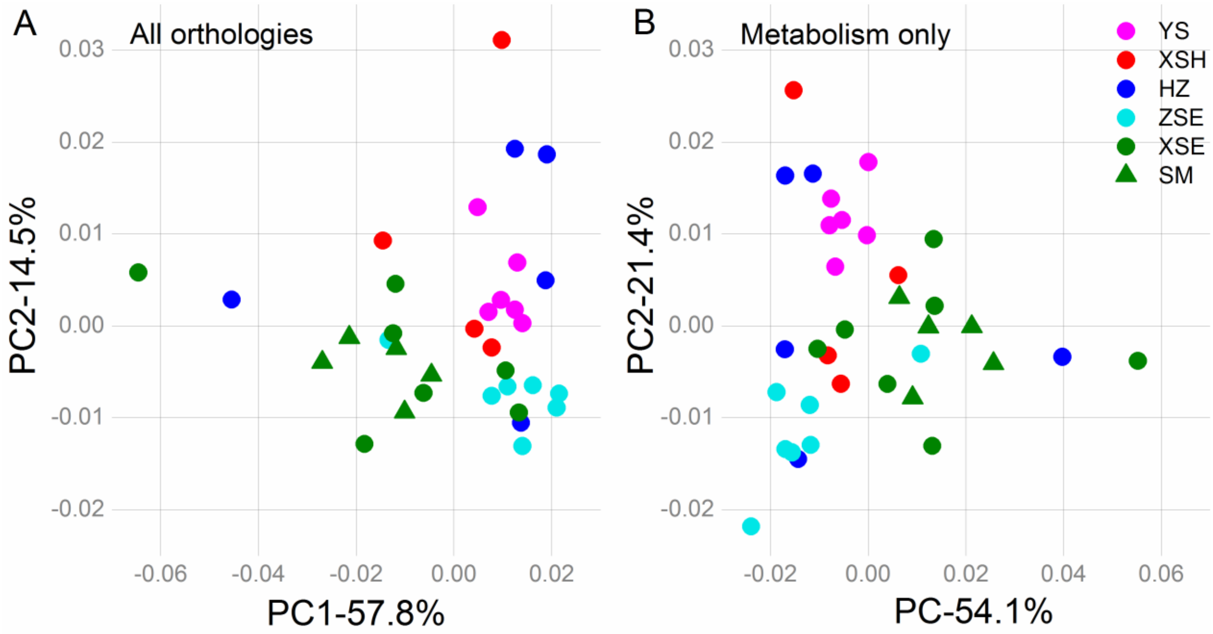

Figure S6. Principal Coordinate Analysis (PCoA) based on Bray-Curtis distance of sediments using (A) PICRUSt predicted KEGG orthologies (KOs) and (B) KOs only classified as "Metabolism" in KEGG functional hierarchies.
